# Effects of a Smoke-Free Policy in Xi'an, China: Impact on Hospital Admissions for Acute Ischemic Heart Disease and Stroke

**DOI:** 10.3389/fpubh.2022.898461

**Published:** 2022-06-21

**Authors:** Meng Chu, Zhiyan Liu, Xinzhu Fang, Yajun Wu, Huannan Liu, Xuan Zhao, Tianxiao Zhang, Qian Wu, Fang Tan

**Affiliations:** ^1^Department of Epidemiology, School of Public Health, Health Science Center, Xi'an Jiaotong University, Xi'an, China; ^2^Department of Respiratory and Critical Care Medicine, Xi'an No.3 Hospital, The Affiliated Hospital of Northwest University, Xi'an, China; ^3^Ministry of Health Education, Xi'an Health Education Institute, Xi'an, China; ^4^Center for Infectious Disease Control and Prevention, Global Health Institute, Health Science Center, Xi'an Jiaotong University, Xi'an, China

**Keywords:** stroke, acute ischemic heart disease, second-hand exposure, tobacco, smoking, smoke-free policy

## Abstract

**Background:**

Smoking and secondhand smoke (SHS) exposure rates are much higher in China than in other countries. A smoke-free policy was implemented in Xi'an, a city in Shaanxi Province, China, on November 1, 2018. This study aimed to evaluate the effect of the smoke-free policy on changes in hospital admissions for acute ischemic heart disease (AIHD) and stroke in Xi'an.

**Methods:**

All subjects had been hospitalized for AIHD or stroke from February 9, 2017 to December 25, 2019 (study period: 150 weeks) in six randomly selected public hospitals out of 36 tertiary hospitals in Xi'an. A generalized additive model developed using an interrupted time series design was used to compare immediate and annual percent changes in hospital admissions before and after policy implementation.

**Results:**

The study included 31,400 cases (16,656 cases of AIHD and 14,744 cases of stroke) from 6 hospitals in Xi'an. Immediately after implementation of the smoke-free policy, AIHD admissions were reduced significantly (−31.66%, 95% CI: – 39.45 to −22.86%), but stroke admissions were not (−4.94%, 95% CI: −13.26 to 4.17%). The annual reduction in stroke-related admissions (−14.54%, 95% CI: −23.53 to −4.49%) and the annual increase in AIHD-related admissions (40.58%, 95% CI: 22.08 to 61.87%) were significant. Although there was no significant reduction in AIHD admissions, stroke admissions were significantly reduced by −15.73% (from 7,350 to 6,194) after implementation of the smoke-free policy in Xi'an.

**Conclusion:**

The smoke-free policy had different effects on hospital admissions for AIHD and stroke in Xi'an. Xi'an should improve its smoke-free legislation and expand the measures to maintain or achieve additional significant health benefits. These findings can guide the formulation and implementation of regional and national smoke-free policies.

## Introduction

In 2019, 341 million (30%) of the 1.14 billion tobacco smokers worldwide were located in China, and the age-standardized rate of smoking in China was 49.7% (318 million) for males and 3.54% (23.2 million) for females (1) In that year, Chinese tobacco consumption was 2,150 cigarette equivalents per person, which was much higher than that worldwide (1,110 per person). Consequently, the smoke-related deaths in China increased from 1.5 million in 1990 to 2.4 million in 2019, and a 57.9% increase in smoke-attributable deaths (1). China has implemented many measures in an effort to reduce smoking rates (i.e., the creation of smoke-free locations, local smoke-free policies), but the effects are very limited (1). Smoke-free policies provide short- and long-term health benefits mainly through reducing exposure to second-hand smoke (SHS) in the environment and reducing active smoking by encouraging cessation of smoking and increasing public awareness about the dangers of smoking (2).

The “Healthy China Program (2019–2030)” has the goal of reducing smoking rates to 24.5 and 20% among people over 15 years old by 2022 and 2030, respectively, and protecting 30 and 80% of the population with comprehensive smoke-free regulations (3). However, as of January 2022, there were only 20 100% smoke-free cities in China, and while 11 cities (Qingdao, Beijing, Lanzhou, Shenzhen, Shanghai, Xi'an, Hangzhou, Qinhuangdao, Wuhan, Zhangjiakou, and Xining) had specific smoke-free laws, this covered only 213 million people (15.1% of the total population) (4). Thus, the smoke-reduction goals seem unlikely to be met on schedule.

Studies on the health effects of a smoking ban on ischemic heart disease (IHD, including acute myocardial infarction, AMI) and cerebrovascular diseases (CVD, including stroke) have very different findings due to differences in the policy details and implementation degrees. For example, Spain's partial and comprehensive smoking ban did not significantly reduce hospitalizations for IHD or CVD (5). The national Irish smoking ban (6) was associated with immediate reductions in IHD (−26%) and stroke (−32%) mortality, but the annual effects were non-significant. The partial smoking ban in Hong Kong (7) significantly reduced the annual mortality due to IHD (−9.3%) but not that due to CVD. Even in the 100% smoke-free cities in China, AMI and stroke hospitalizations in Beijing (8) and Qingdao (9) still greatly differed in immediate and annual changes. Specifically, in Beijing, the stroke hospitalizations significantly decreased both immediately and annually, while AMI decreased only immediately; in Qingdao, the stroke hospitalization significantly decreased in annually, but AMI hospitalizations increased immediately with an annual decrease.

Xi'an implemented a comprehensive smoking ban on November 1, 2018 (10) However, the actual health effects of the smoking ban are still unclear. This study examined hospital admissions for acute ischemic heart disease (AIHD) and stroke in Xi'an before and after policy implementation to evaluate the health effects of the ban, fill gaps in the impact of smoking bans in Xi'an, and provide evidence for policy-makers to further improve and enforce measures in the future. We also hope that this study will provide reliable evidence for promoting national smoke-free legislation in China and provide clues to other countries.

## Materials and Methods

### Materials

According to data accessibility, we selected 6 tertiary hospitals in Xi'an, accounting for about 16.67% of the tertiary hospitals. The study subjects were patients hospitalized for AIHD or stroke from February 9, 2017 to December 25, 2019, for a total of 150 weeks. Data were obtained from the electronic case registration systems of the 6 hospitals and determined based on the first discharge diagnosis according to the International Classification of Diseases, 10th revision (ICD10): AIHD (I20–I24) and stroke (I60–I64). The extracted information included age, sex, birth place, current residence, work address, admission time, discharge department, main discharge diagnosis code, main discharge diagnosis name, etc. Patients under the age of 15 years or who were not permanent residents of Xi'an (11) were excluded from this study. Patients who were admitted to the hospital repeatedly for the same disease within 28 days were also excluded; only the first admission within this period was retained.

Data on air pollution in Xi'an were obtained from the official website of the Shaanxi Provincial Department of Ecology and Environment (http://sthjt.shaanxi.gov.cn/), and the 24-h average temperatures (°C) and relative humidity (%), which represent the average levels in Xi'an, were obtained from the China Meteorological Data Sharing Service System (http://data.cma.cn/). Annual legal holidays (New Year's Day, Spring Festival, Qingming Festival, Labor Day, Dragon Boat Festival, Mid-Autumn Festival, and National Day) were determined according to the information issued by the General Office of the State Council of the People's Republic of China (http://www.gov.cn/zhengce/xxgk/index.htm). The population over 15 years of age in Xi'an was calculated based on the 2018–2020 Statistical Yearbook of Xi'an (http://tjj.xa.gov.cn/); this value was used to calculate the hospital admission rate and was an offset variable in the model to adjust for possible changes in the population over time. Ethical approval was granted by the Biomedical Ethics Committee of Xi'an Jiaotong University (No: 2020-1350).

### Statistical Analysis

The outcomes of this study were the number of admissions for AIHD and stroke each week, and data spanned multiple time points before and after the intervention. Policy studies usually use an interrupted time series (ITS) design to assess whether “changes” in outcomes are attributable to the intervention by comparing the immediate and annual relative changes in outcomes following the intervention. Therefore, to test whether hospital admission rates for AIHD and stroke changed after the policy implementation, a generalized additive model (GAM) was developed using the ITS design. All variables in the model were analyzed as a time series in weeks. The cubic spline function was used to fit non-linear parameters such as the air quality index, average temperature and relative humidity. The number of legal holidays per week was also included in the model to illustrate differences between weeks. A preliminary analysis showed that the long-term trend was linear. Therefore, time was included in the model as a linear variable to quantify the changes in population risk factors and other long-term trends. In addition, an interaction between the tobacco control policy and the time after the policy was implemented was also included in the model to estimate the change in the long-term trend slope between the pre- and post-policy periods. Seasonality was modeled by a Fourier series of sine and cosine terms (11).

The immediate relative change (percentage) in hospital admissions from the pre- to post-policy period was calculated as 100 (exp(β_2_)-1), which represents the change in admissions in a given week, and the annual relative change was calculated as 100(exp ($52^{*}\beta_{3}$) −1) (11). The number of people protected by the policy was defined as the difference between the number of admissions predicted by the model and the actual number of admissions. To assess the impact of the policy on different population subgroups, the data were stratified by age (<65 years old or ≥65 years old) and sex and analyzed in each subgroup. To further verify whether the final model detected the real effects of the smoke-free policy, a series of false intervention dates were created to test the robustness of the model. All analyses were conducted in R (V.3.5.1), (12) and the model was carried out with the “mgcv” package (13).


$$\begin{array}{l} log\left(Y\right)=offset\log\left(P\right)+\beta_{0}+\beta_{1}T+\beta_{2}Int+\beta_{3}(Int\;*\;T^{'})\\ \;+\beta_{4}\cos(\frac{2T\;*\;\pi }{52})+\beta_{5}\sin(\frac{2T\;*\;\pi }{52})\\ \;+\varepsilon +Holiday+s(X^{\prime }) \end{array}$$

Where Y represents the weekly outcome variable, P represents the average population, and T represents the time since the start of the study; Int is a dummy variable representing whether the policy was implemented (before the policy was implemented, the value was 0, after the policy was implemented, the value was 1); T' represents the time after the smoke-free policy was implemented; β_0_ represents the baseline level at T = 0, β_1_ represents the model coefficient of the pre-policy time trend, β_2_ is the coefficient when the intervention variable (policy) was implemented, indicating the immediate change of the policy, and β_3_ indicates the long-term slope change following implementation of the smoke-free policy; sin/cos is the seasonal factor adjusted according to the Fourier function; Holiday is the statutory holiday; *s* is a smooth function; and *X'* are nonlinear parameters including air quality index, weekly average temperature and average relative humidity.

## Results

From February 9, 2017 to December 25, 2019, 31,400 cases (16,656 cases of AIHD and 14,744 cases of stroke) were admitted to 6 hospitals in Xi'an. The specific numbers of hospital admissions by sex and age subgroups are shown in Table 1. Males and elderly people (≥65 years old) had a larger number of hospital admissions for these two diseases than females and younger people. In terms of the average annual crude admission rates, stroke and AIHD admission rates in men were significantly higher than those in women in Xi'an.

**Table 1:** Descriptive characteristics of AIHD and stroke hospital admissions in Xi'an by age and sex, *n* (%).

	**Stroke**		**AIHD**	
**Age**	**Male**	**Female**	**Male**	**Female**
	**(*n* = 9,529)**	**(*n* = 5,215)**	**(*n* = 9,261)**	**(*n* = 7,395)**
≤ 64 years	4,125 (43.30)	1,607 (30.80)	4,805 (51.90)	2,719 (36.80)
≥65 years	5,404 (56.70)	3,608 (69.20)	4,456 (48.10)	4,676 (63.20)
*χ^2^*	220.70		379.35	
*P*	**<0.001**		**<0.001**	

*Bold indicates P value <0.05, which is statistically significant. AIHD, acute ischemic heart disease*.

**Figure 1:**
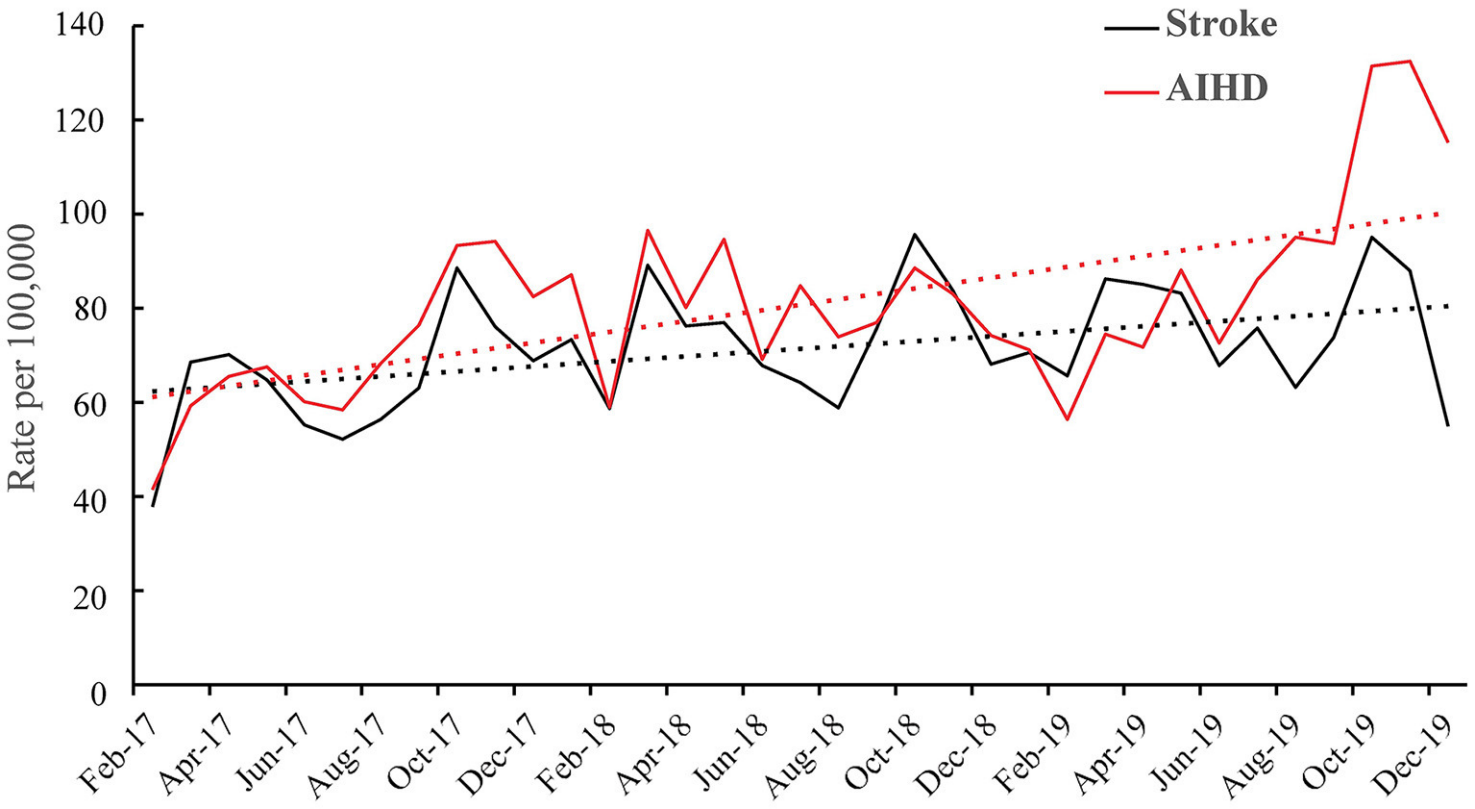
Monthly hospital admission rates for AIHD and stroke and fitted linear trends from February 2017 to December 2019. The solid line represents the actual monthly admission rates, and the dashed line represents the fitted linear trend. Black represents stroke, and red represents AIHD. AIHD, acute ischemic heart disease.

Figure 1 shows the monthly trends of hospital admission rates for AIHD and stroke. The rates were higher for AIHD, and the predicted trends increased markedly. As shown in Figure 1, there were seasonal patterns for the two diseases: the AIHD and stroke admission rates from October to December were much higher than those in other months.

### Changes in Hospital Admissions

**Figure 2:**
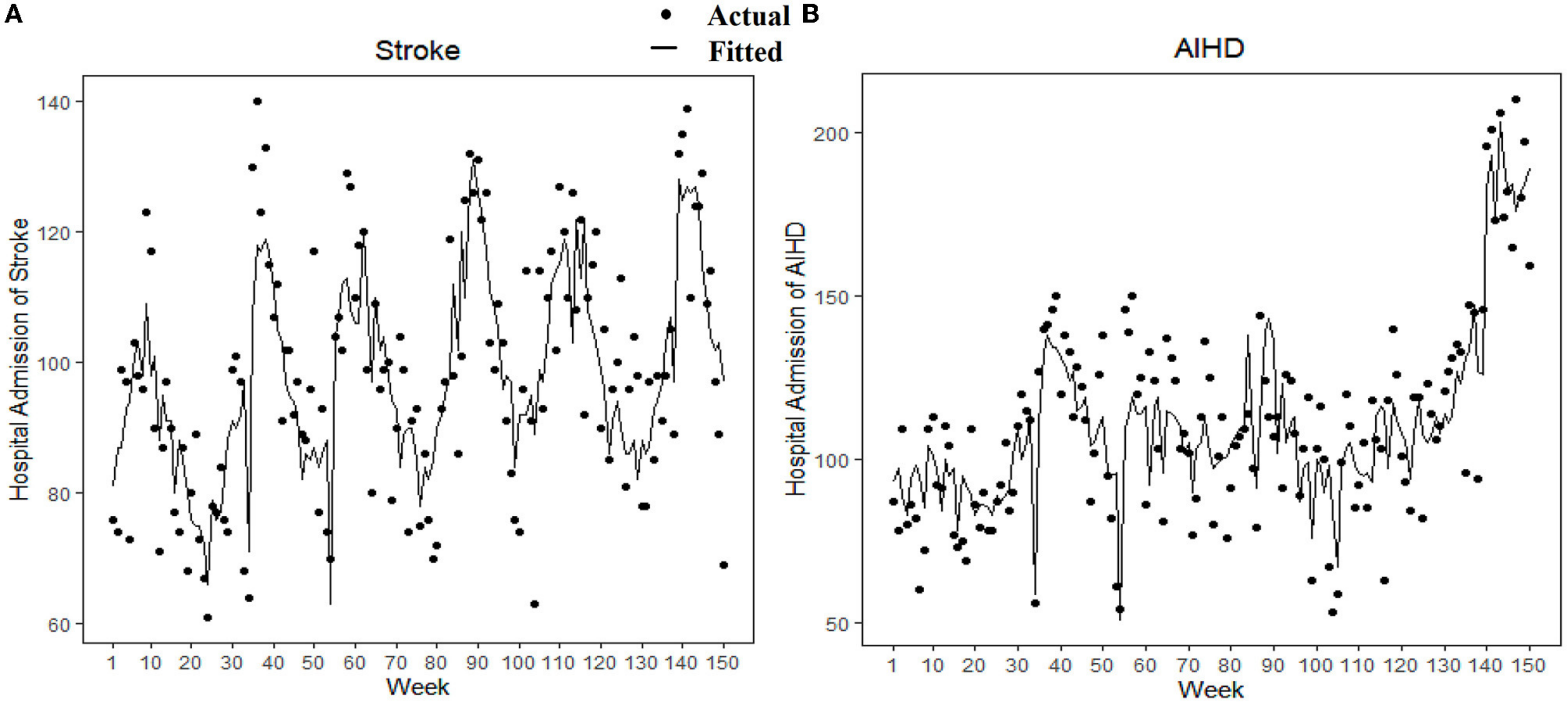
The actual distribution and GAM model fitting trends of weekly hospital admissions for stroke and AIHD. Points indicate the actual number of hospital admissions per week, and the line represents the number of weekly hospitalizations fitted by the GAM model. AIHD, acute ischemic heart disease; GAM: Generalized additive model.

Figure 2 shows the model-fitted trends of the actual number of weekly hospitalizations for stroke and AIHD; the sex- and age-specific group trends are shown in Supplementary Figures 1, 2. The changes in hospital admissions for AIHD and stroke after implementation of the smoke-free policy in Xi'an on November 1, 2018 are shown in Table 2, with the set of adjusted variables. The immediate reduction in hospital admissions was approximately −31.66% (95% CI: – 39.45 to −22.86%) for AIHD, while the immediate reduction for stroke was non-significant (−4.94%, 95% CI: −13.26 to 4.17%). Additionally, regarding annual trends, the change in stroke hospital admissions was −14.54% (95% CI: −23.53 to −4.49%), while AIHD hospital admissions significantly increased by 40.58% (95% CI: 22.08 to 61.87%) (Table 2). The average percentage change in hospital admissions for the two diseases on a semester basis is provided in Supplementary Table 1.

**Table 2:** Average percentage change^*^ (%) in hospital admission rates of all groups and sex- and age-specific subgroups due to the smoke-free policy in Xi'an, 2017–2019.

	**Stroke**		**AIHD**	
	**Immediate change (95%CI)**	**Annual change (95%CI)**	**Immediate change (95%CI)**	**Annual change (95%CI)**
Overall	−4.94 (−13.26 to 4.17)	**−14.54 (−23.53 to** **−4.49)**	**−31.66 (−39.45 to** **−22.86)**	**40.58 (22.08 to 61.87)**
<65years	−8.3 (−18.43 to 3.09)	**−14.58 (−25.92 to** **−1.49)**	**−33.65 (−43.03 to** **−22.73)**	**49.39 (24.99 to 78.56)**
≥65years	−4.72 (−14.79 to 6.54)	**−16.89 (−27.41 to** **−4.83)**	**−29.72 (−37.98 to** **−20.36)**	**31.17 (13.39 to 51.74)**
Male	−2.78 (−12.53 to 8.06)	**−14.53 (−24.67 to** **−3.04)**	**−30.15 (−38.82 to** **−20.25)**	**39.15 (19.19 to 62.45)**
Female	−11.33 (−22.19 to 1.04)	−14.73 (−27.37 to 0.1)	**−33.5 (−42.81 to** **−22.67)**	**44.04 (20.64 to 71.97)**

Bold indicates P value <0.05, which is statistically significant. *, Adjusted for air quality index, legal holidays, population, seasonality, time trend, temperature and relative humidity. AIHD, acute ischemic heart disease; CI, confidence interval.

The effects of the policy on the different sex and age groups are shown in Table 2. The hospital admissions for stroke did not immediately decrease significantly in any subgroup. Similarly, the annual female admissions for stroke did not significantly decrease (*P* > 0.05). The immediate AIHD admissions in each subgroup decreased significantly, while the annual AIHD admissions increased significantly.

We conducted additional sensitivity analyses to further verify our results. Overdispersion was found to be very limited, and we adjusted for it by following a “quasi-Poisson” distribution. Several false implementation dates were applied to test the impact of the policy on hospital admissions for the two diseases. We found that the false date results were not significant and that the magnitude of the change was smaller than the actual immediate change (Supplementary Table 2).

### Protective Effect of the Policy Against Hospital Admissions

**Figure 3:**
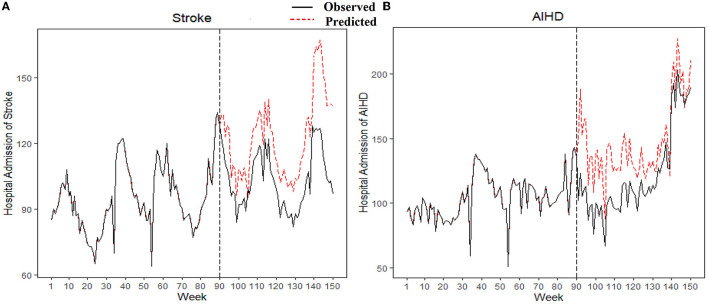
The predicted and observed hospital admissions for stroke and AIHD from February 2017 to December 2019. The red line represents the predicted hospital admissions without the smoke-free policy according to the GAM model; the black line represents the observed hospital admissions. AIHD, acute ischemic heart disease; GAM, Generalized additive model.

As shown in Figure 3, the protective effect of the policy was calculated as the difference between the predicted (red line in Figure 3) and observed hospital admissions (black line in Figure 3). The predicted and observed numbers of hospital admissions in the sex- and age-specific groups are shown in Supplementary Figures 3, 4. If the smoke-free policy was not implemented, hospital admissions for stroke and AIHD from November 1, 2018 to December 25, 2019 would have reached 7,350 (95% CI: 6362, 8,337) and 8,813 (95% CI: 7,258, 10,374), respectively. However, the actual hospital admissions for stroke and AIHD during this period were 6,194 and 7,273, respectively. Although there was no significant reduction in AIHD admissions, stroke admissions in Xi'an were reduced by −15.73% due to the smoke-free policy.

## Discussion

This study is the first to evaluate the comprehensive smoke-free policy in Xi'an. We randomly selected 6 hospitals with complete electronic medical record system data based on their geographic location. The dataset contained 150 time points, including 90 weeks before the policy was implemented. We excluded nonresident hospital admissions and adjusted for the air quality index, average temperature and humidity, legal holidays, time trends, seasonal trends, and possible population changes during this period. After analyzing 31,400 hospitalized patients in Xi'an from 2017 to 2019, we found that the implementation of the smoke-free policy in Xi'an differentially impacted AIHD and stroke in patients over 15 years old.

An exposure to tobacco smoke lasting between 20 min and 8 h increases platelet sensitivity and reduces the ability of the heart to receive and process oxygen. Long-term exposure to tobacco smoke can cause coronary vasospasm through abnormal vascular activity, thereby inducing cardiovascular and cerebrovascular diseases (14, 15) Therefore, smoke-free policies can prompt not only short-term effects but also long-term health benefits (2) In Xi'an, the smoke-free policy clearly states that smoking is prohibited in indoor public places, public transport and outdoor areas in some public places (10).

The immediate changes in hospitalizations for stroke after implementation of the smoke-free policy were nonsignificant; however, the annual trend of stroke admissions decreased significantly (−14.54%, 95% CI: −23.53 to−4.49%). This result was consistent with those of studies in Beijing, (8) Qingdao, (9) and Arizona; (16) in contrast, studies in Chile (17) and the United States (18) showed no significant impact on stroke admissions. The disease burden caused by stroke in China is much higher than that in other countries; therefore, the health benefits of smoke-free policies on stroke may be more obvious in China (19, 20).

After Xi'an's implementation of the smoke-free policy, significant immediate decreases in AIHD admissions (−31.66%, 95% CI: – 39.45 to −22.86%) were observed, while the annual AIHD admissions increased significantly (40.58, 95% CI: 22.08 to 61.87%). The immediate trend was consistent with that of studies in Beijing (8) and Ireland (6). However, the significant increase in the annual trend differed from the results of studies conducted in Germany, (21) Uruguay, (22) Spain (23) and Qingdao (9). These studies found that the annual cardiovascular disease admissions were reduced due to the implementation of smoke-free laws.

The immediate effects of smoking on IHD are greater and more drastic than those on stroke. A comparison of current smokers with non-smokers revealed that the hazard ratio (HR) for IHD is higher than that for stroke (1.60 vs. 1.32, respectively), (24) and even non-smokers who are exposed to SHS at home and at work have much higher risks of IHD than non-smokers not exposed to SHS (25 vs. 20%, respectively) (25). After a smoker quits smoking, their risk of cardiovascular disease begins to decrease almost immediately (26). Compared with current smokers, the HRs associated with ex-smokers were 0.71 for IHD and 0.84 for stroke (24).

Smoking is one of the most common risk factors for stroke in China, accounting for a relatively high proportion (31.7–47.6%) of stroke incidences and ranking 2^nd^ among all risks (27). Therefore, the health effects of stroke may be more obvious than those of IHD, and the significant decrease in the annual stroke admissions also indicates the effectiveness of the tobacco control measures in Xi'an (28). However, the annual IHD admissions continued to increase, which may be related to the small proportion of tobacco in the etiology of IHD and continuous increases in other risk factors (29). High systolic blood pressure, high total cholesterol, diets high in sodium, diets low in whole grains and smoking were the five most common risk factors for IHD in China. Although smoking dropped from 4^th^ place in 1990 to the 5^th^ place in 2015, representing the largest decline in age-standardized IHD disability-adjusted life year (DALY) rates among these factors (−14.5%), the DALY rates of high systolic blood pressure and diets high in sodium continued to increase (30 and 4.1%, respectively) (29) Moreover, as independent risk factors for IHD, these factors may have synergistic effects with smoking (30, 31) and obscure the effects of smoke-free policies. (32) Residents of Xi'an, the capital of Shaanxi Province, have diets characterized by high sodium and low potassium contents, with daily sodium intake far exceeding the 5 g recommended by the World Health Organization (32) However, increasing the amount of potassium in salt and reducing sodium provides the greatest protection against CVD, a level of protection greater even than quitting smoking (32) In 2015, the prevalence of hypertension in Xi'an was 41.59%, and the dyslipidemia rate of people over 40 years old was 50.34%, both higher than the national average, especially for women (30, 31) With the accelerated aging process, the standardized mortality rate and number of deaths due to IHD in Shaanxi Province increased by 34.5 and 177.5%, respectively, from 1990 to 2015 (29) Therefore, it is difficult to reverse the continuous increase in IHD admissions in Xi'an by relying on only tobacco control policies. We even suspect that without the implementation of the tobacco control policy, the IHD admissions in Xi'an would be much higher.

Moreover, the differences in the annual trends may reflect the incomplete implementation of Xi'an's smoke-free policy, which has caused a rebound effect. A survey conducted in Xi'an in April 2019 found that the PM_2.5_ and nicotine concentrations in restaurants were not significantly reduced (33) and that the concentrations were still higher than those in Beijing, (34) Hangzhou, (33) New Zealand (35) and Latin America, (36) indicating that after policy implementation, the rate of exposure to SHS in Xi'an restaurants was higher than those in other domestic cities and other countries.

The smoke-free policies implemented in different regions result in large differences in health effects according to age (7, 8) and sex. The main reasons for these differences may be related to differences in social behaviors, smoking rates, SHS exposure rates or policy implementation among these groups (8). There were no significant differences in the immediate stroke admissions among the subgroups. However, the annual changes in stroke admissions were significant in all subgroups except the female subgroup. The immediate and annual changes in AIHD admissions were significant in both the age and sex subgroups, with a greater impact on the population under 65 and women. The results are consistent with those of studies in Kentucky, (37) Beijing (38) and Italy (39). Many studies have indicated that although there is no sex difference in the effects of smoking on stroke, female smokers have a 25% higher risk of IHD than men (24, 25). This may be related to the effect of smoking on menopause and the antiestrogen effects of smoking. (24, 40). Long-term heavy smoking has a greater impact on aortic sclerosis in women than in men (40) Data from Shaanxi Province (41) have shown that both the SHS exposure rate and the female smoking rate increased from 2010 to 2015 (49.46 to 65.79% and 1.60 to 2.28%, respectively). Moreover, the SHS exposure rates in women and people <65 years old were higher than those in other populations (41, 42) The opposite AIHD trends in the subgroups also indicate that tobacco control policies were effective but have not been completely implemented. Female smoking is generally more harmful to family health than male smoking (24) Even in elderly women with definitive IHD, smoking cessation can improve survival and reduce reinfarction rates (40). Given the increasing smoking rate among women in Shaanxi Province and the low smoking cessation rate, (41) more attention should be given to female smokers during the implementation of tobacco control policies.

By September 30, 2019, more than 2,69,000 locations were subject to supervision and inspection in Xi'an, and more than 50,000 individuals and 21,000 units were ordered to rectify breaches in conduct (4) An investigation of SHS exposure in some indoor restaurants in Xi'an in April 2019 found that the number of posted non-smoking signs and the smoking incidence improved significantly after the policy was implemented (33) However, levels of fine particulates (PM_2.5_) and nicotine in restaurants showed non-significant decreases (from 52.00 to 37.00 ug/m^3^ and from 4.47 to 3.04 ug/m^3^, respectively), (33) and concentrations of nicotine and SHS exposure in men's restrooms and private rooms of indoor restaurants (33) were much higher than those in Beijing (34) and Hangzhou (33) Therefore, the government of Xi'an still needs to further improve the implementation of this policy to expand the smoke-free environment.

Compared with other cities' smoke-free policies, (43, 44) the current smoke-free policy in Xi'an only bans tobacco, (10) not e-cigarettes; thus, other tobacco control measures also need to be improved (such as banning cigarette and e-cigarette advertising and the sale of cigarettes and e-cigarettes to minors on campuses) (44). However, the most critical aspect is that the punishment for illegal actions, both of individuals and places, is too mild (10, 43) (individuals [Xi'an vs. Beijing]:10 vs. 50–200 Chinese yuan [CNY] fine; units [Xi'an vs. Beijing]: 500–1,000 vs. 2,000–10,000 CNY fine) and does not deter smoking, which to some extent weakens the effectiveness of law enforcement. Thus, it is hoped that these issues will be addressed in the newly revised law.

This study has several limitations. First, our study did not verify the implementation of the smoke-free policy and the SHS exposure levels in Xi'an. Second, the disease type was determined by only the first discharge diagnosis, and patients who were not admitted to the hospital were not considered (such as those who died outside the hospital). Additionally, there are complex and close relationships between the incidence of AIHD and stroke, and it was difficult to identify misdiagnoses in the data given the existing data and resources. However, a study found that the estimated rates of IHD and stroke were only slightly affected due to the compensatory methods of misclassification at the population level (11). Moreover, the misclassification of the above two diseases is likely to have nothing to do with the implementation of the smoke-free policy, so it may not have significantly impacted the results. Third, regarding the availability of data, the patients in this study were from tertiary hospitals with relatively complete electronic medical records systems, and there may have been bias in the results. In addition, although potential time trends and other covariates were adjusted for in the analysis, due to the lack of information, some important variables were not controlled for, such as education level, obesity, population cholesterol levels, and influenza outbreaks.

The more comprehensive a law is, the greater its impact (45). However, China has made little progress in reducing smoking rates or introducing national smoke-free laws (1). Hundreds of millions of people will be protected from the dangers of SHS if comprehensive smoke-free laws are effectively enforced and indoor exposure is eliminated, preventing 6 million premature deaths related to smoking (46). Before the realization of national smoke-free legislation, Xi'an should continue to improve policy implementation, expand policy scope, and improve the environment by reducing SHS exposure.

## Conclusions

The smoke-free policy in Xi'an had different effects on AIHD and stroke hospital admissions. The changes in AIHD admissions may have been affected by various factors, such as incomplete implementation of the policy and related risks or diseases. Xi'an should continue to improve its smoke-free legislation and expand the scope of implementation to maintain or achieve additional significant health benefits. These findings are also important for the future formulation and implementation of regional and national smoke-free policies.

## Data Availability Statement

The raw data supporting the conclusions of this article will be made available by the authors, without undue reservation.

## Ethics Statement

The studies involving human participants were reviewed and approved by Biomedical Ethics Committee of Xi'an Jiaotong University (No: 2020-1350). Written informed consent for participation was not required for this study in accordance with the national legislation and the institutional requirements.

## Author Contributions

QW, XF, FT, and ZL contributed to the study concept and had full access to all the data in the study. MC, YW, and HL were responsible for the integrity of the data. MC interpreted the findings and drafted the article. MC and TZ contributed to the data analysis. MC and ZL interpreted the data. All authors critically revised and approved the final manuscript.

## Funding

This research was supported by the Shaanxi Xinhang Public Health Research Center (20201053).

## Conflict of Interest

The authors declare that the research was conducted in the absence of any commercial or financial relationships that could be construed as a potential conflict of interest.

## Publisher's Note

All claims expressed in this article are solely those of the authors and do not necessarily represent those of their affiliated organizations, or those of the publisher, the editors and the reviewers. Any product that may be evaluated in this article, or claim that may be made by its manufacturer, is not guaranteed or endorsed by the publisher.
