# Supplementary material for: Effects of a Smoke-Free Policy in Xi'an, China: Impact on Hospital Admissions for Acute Ischemic Heart Disease and Stroke
Source: Front Public Health. 2022 Jun 21;10:898461. doi: 10.3389/fpubh.2022.898461 (PMC9253815; doi:10.3389/fpubh.2022.898461)
Supplement: Supplementary file 1 [file Data_Sheet_1.docx]

**Supplementary Table 1**. Immediate and Semester average percentage change* (%) in hospital admission rates of all groups and sex- and age-specific subgroups due to the smoke-free policy in Xi’an, 2017–2019

|  | Stroke | | | AIHD | |
| --- | --- | --- | --- | --- | --- |
|  | Immediate change(95%CI) | Semester change in the secular trend (95%CI) | Immediate change(95%CI) | | Semester change in the secular trend (95%CI) |
| Overall | -4.94(-13.26 to 4.17） | **-7.55(-12.55 to -2.27)** | **-31.66(-39.45 to -22.86)** | | **18.56(10.49 to 27.23)** |
| <65years | -8.3(-18.43 to 3.09) | **-7.57(-13.93 to -0.75)** | **-33.65(-43.03 to -22.73)** | | **22.23(11.8 to 33.63)** |
| ≥65years | -4.72(-14.79 to 6.54) | -**8.83(-14.8 to -2.45)** | **-29.72(-37.98 to -20.36)** | | **14.53(6.48 to 23.18)** |
| Male | -2.78(-12.53 to 8.06) | **-7.55(-13.21 to -1.53)** | **-30.15(-38.82 to -20.25)** | | **17.96(9.18 to 27.45)** |
| Female | -11.33(-22.19 to 1.04) | -7.66(-14.78 to 0.05) | **-33.5(-42.81 to -22.67)** | | **20.02(9.84 to 31.14)** |

Note: Bold indicates *P* value <0.05, which is statistically significant. *: Adjusted for air quality index, legal holidays, population, seasonality, time trend, temperature and relative humidity. Abbreviation: AIHD: acute ischemic heart disease; CI: confidence interval.

**Supplementary Table 2**: Sensitive analysis of relative risk (RR)* of false smoke-free policy date

| Date | Week in time series | Stroke | | | AIHD | | |
| --- | --- | --- | --- | --- | --- | --- | --- |
|  |  | RR | Lower 95%CI | Upper 95%CI | RR | Lower 95%CI | Upper 95%CI |
| Nov 1, 2018 | 91 | 0.95 | 0.87 | 1.04 | **0.68** | **0.61** | **0.77** |
| False date |  |  |  |  |  |  |  |
| Sep 14, 2017 | 32 | 1.09 | 0.97 | 1.23 | 1.04 | 0.88 | 1.24 |
| Dec 14, 2017 | 45 | 1.04 | 0.93 | 1.16 | 0.95 | 0.81 | 1.12 |
| Feb 22, 2018 | 55 | 0.96 | 0.86 | 1.06 | 0.87 | 0.74 | 1.02 |

Note: Bold indicates *P* value <0.05, which is statistically significant. *: Adjusted for air quality index, legal holidays, population, seasonality, time trend, temperature and relative humidity. Abbreviations: AIHD: acute ischemic heart disease; CI: confidence interval; RR: relative risk.

**Supplementary Figure 1.** The actual distribution and GAM model fitting trends of weekly hospital admissions for stroke and AIHD by gender (A & B for male, C & D for female)

**
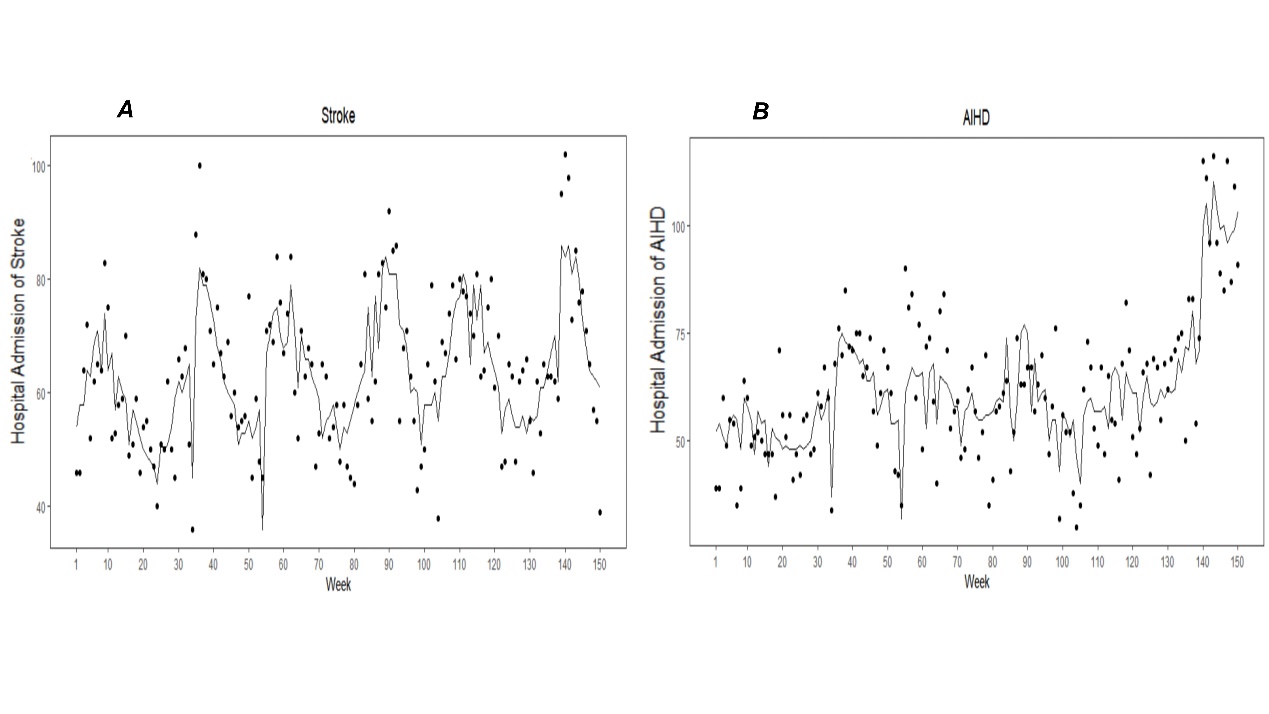
**

**
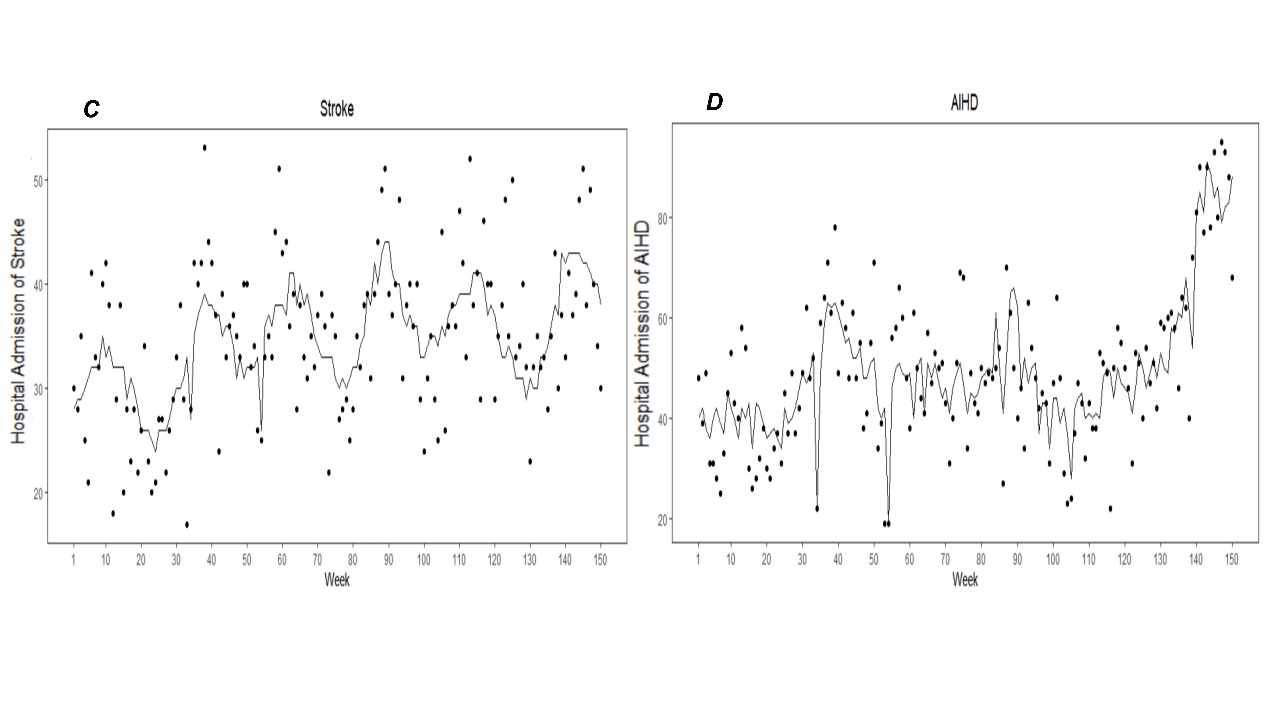
**

Note: Points indicate the actual number of hospital admissions per week, and the line represents the number of weekly hospitalizations fitted by the GAM model.

Abbreviations: AIHD, acute ischemic heart disease; GAM: Generalized additive model.

**Supplementary Figure 2.** The actual distribution and GAM model fitting trends of weekly hospital admissions for stroke and AIHD by age (A & B for ≤64 years, C & D for ≥65 years)


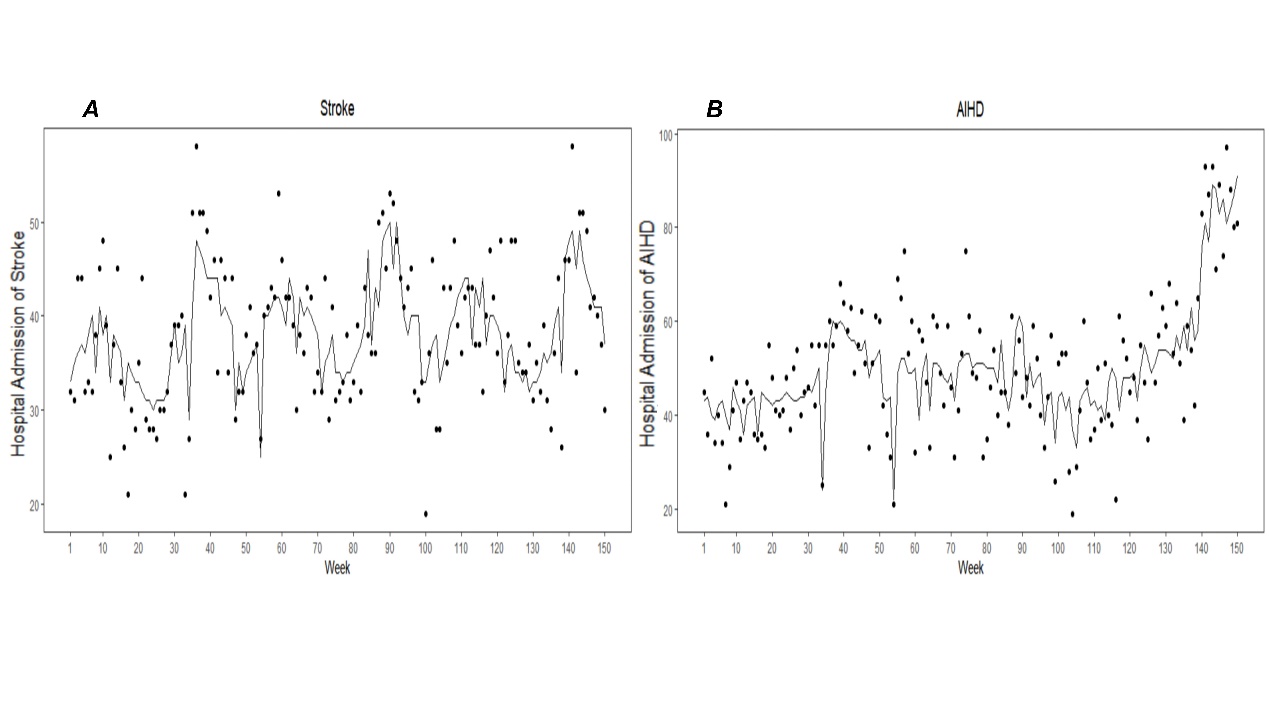
**
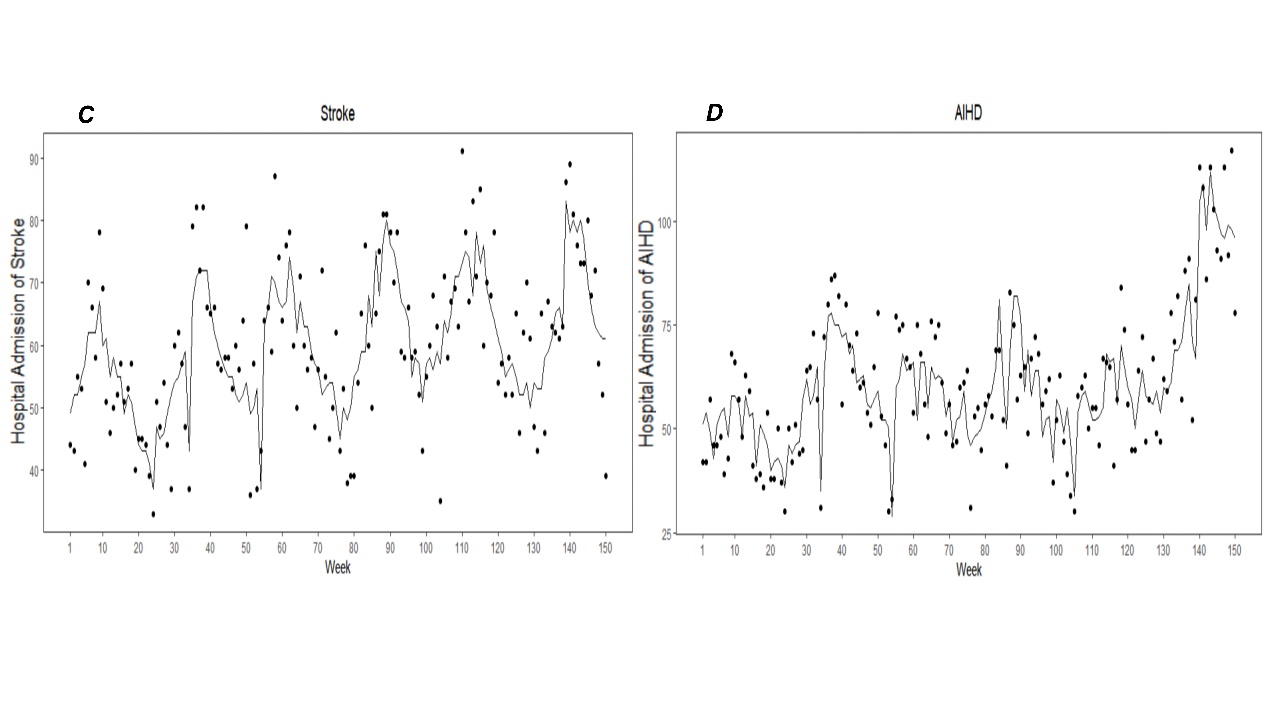
**

Note: Points indicate the actual number of hospital admissions per week, and the line represents the number of weekly hospitalizations fitted by the GAM model.

Abbreviations: AIHD, acute ischemic heart disease; GAM: Generalized additive model.

**Supplementary Figure 3.** The predicted and observed hospital admissions for stroke and AIHD from February 2017 to December 2019 by gender (A & B for male, C & D for female)

**
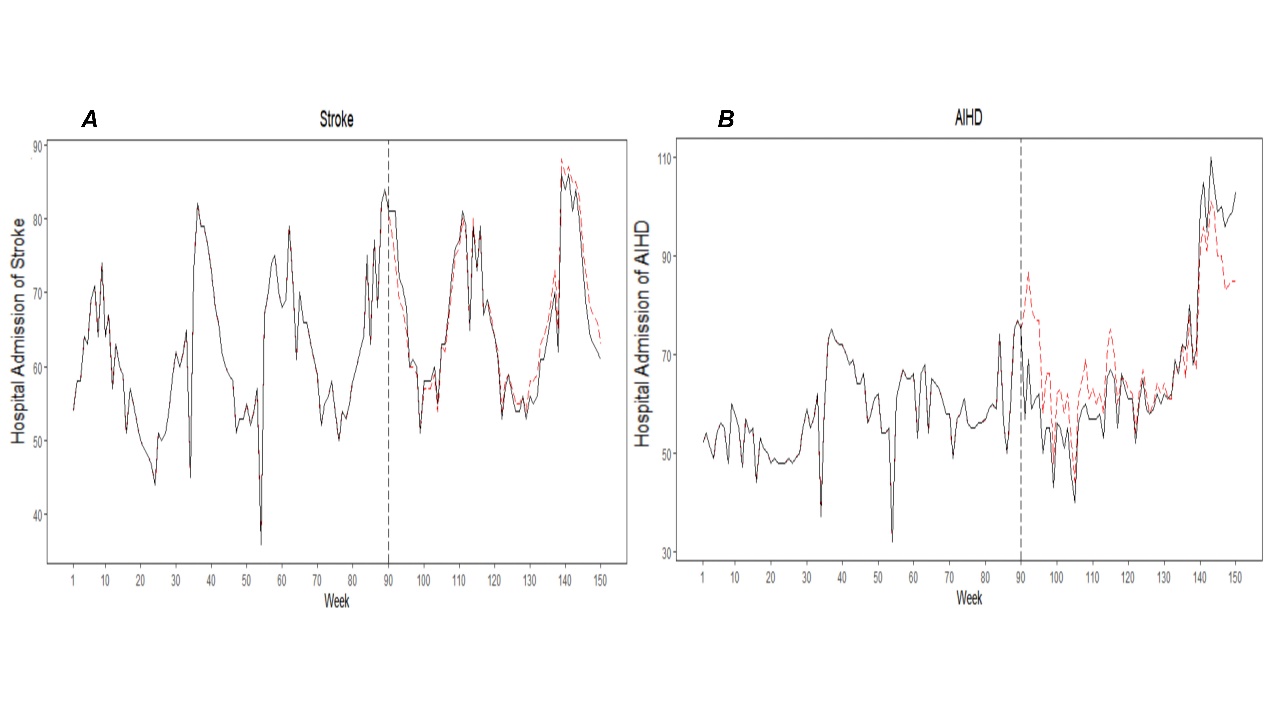
**

**
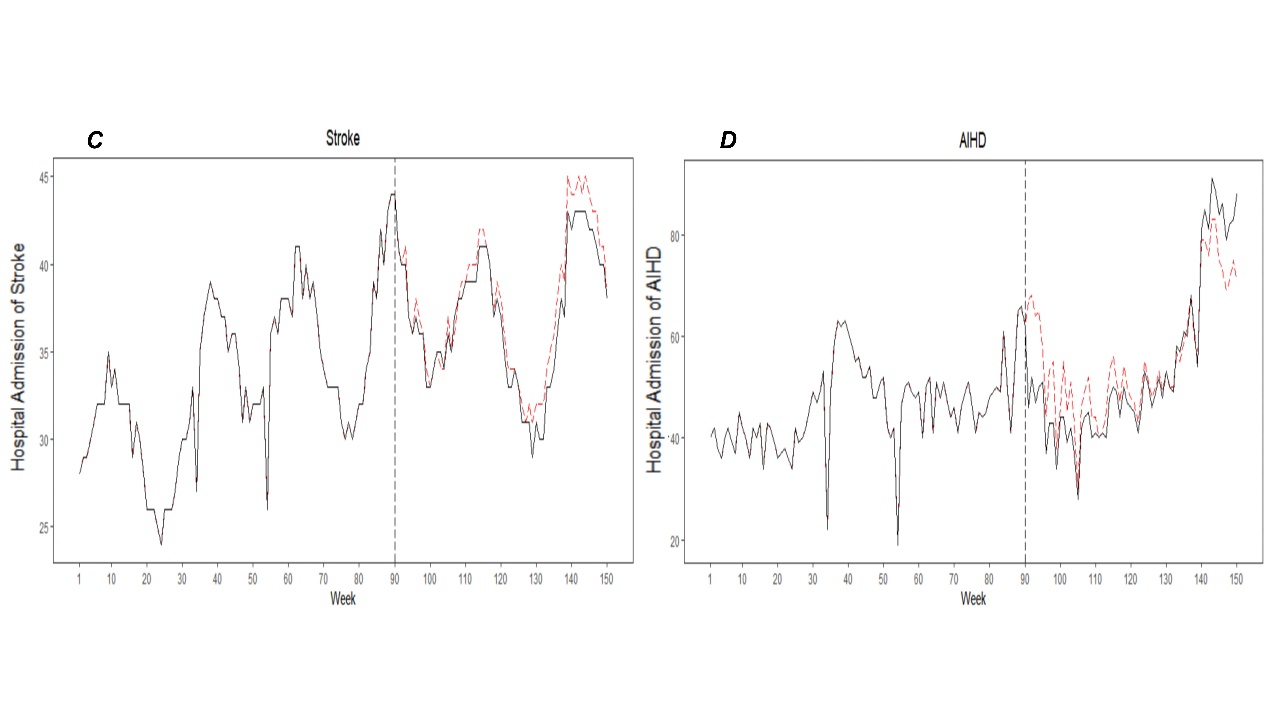
**

Note: The red line represents the hospital admissions without the smoke-free policy according to the GAM model; the black line represents the observed hospital admissions.

Abbreviations: AIHD, acute ischemic heart disease; GAM: Generalized additive model.

**Supplementary Figure 4.** The predicted and observed hospital admissions for stroke and AIHD from February 2017 to December 2019 by age (A & B for ≤64 years, C & D for ≥65 years)

**
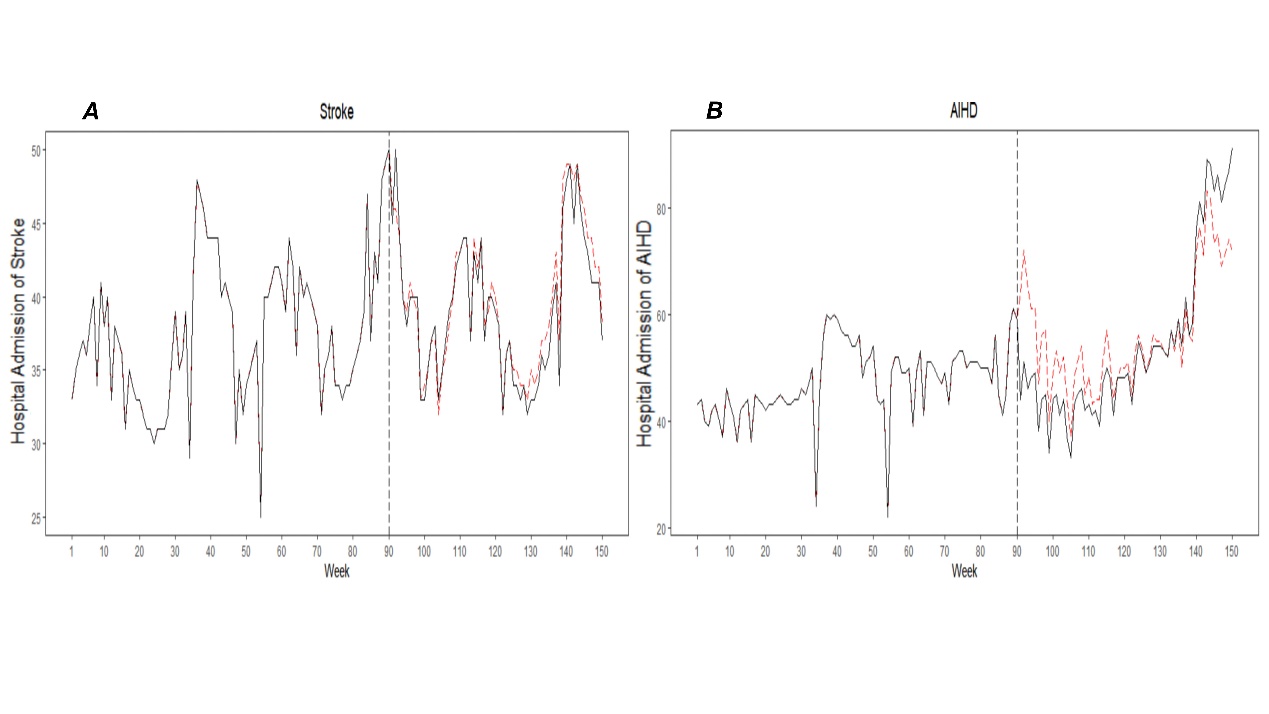
**

**
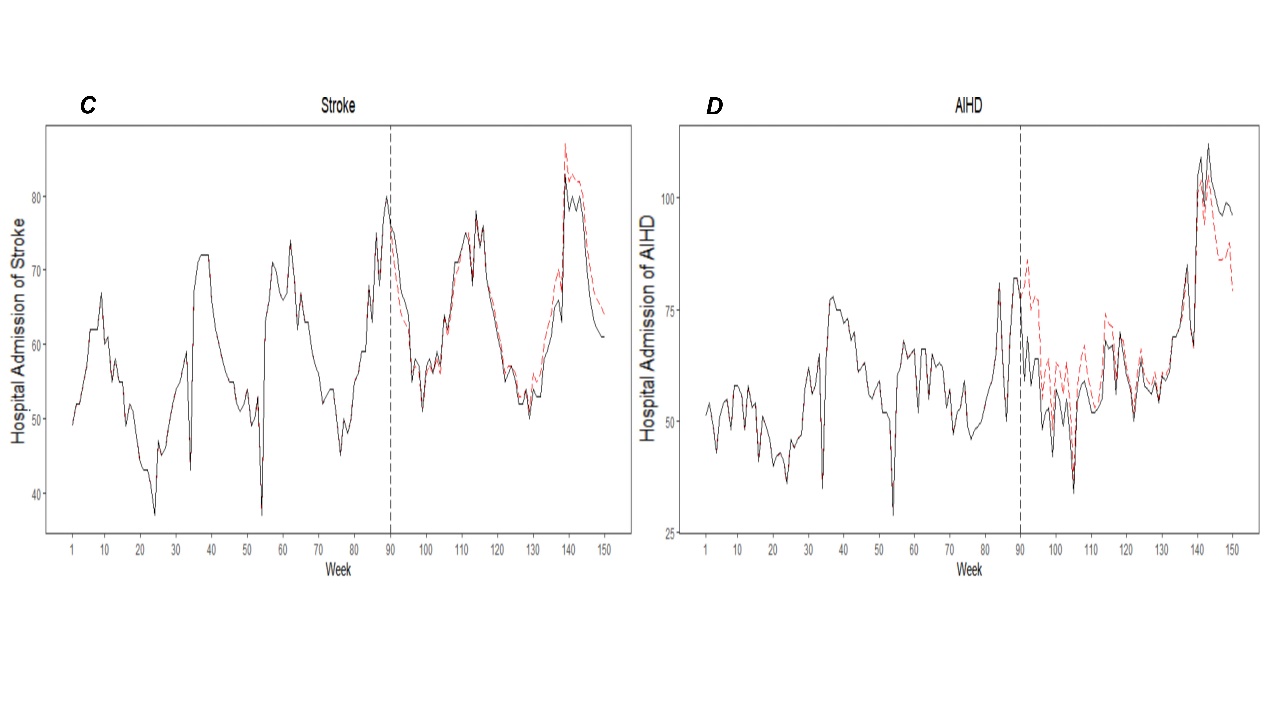
**

Note: The red line represents the hospital admissions without the smoke-free policy according to the GAM model; the black line represents the observed hospital admissions.

Abbreviations: AIHD, acute ischemic heart disease; GAM: Generalized additive model.
